# Adult male circumcision as an intervention against HIV: An operational study of uptake in a South African community (ANRS 12126)

**DOI:** 10.1186/1471-2334-11-253

**Published:** 2011-09-26

**Authors:** Pascale Lissouba, Dirk Taljaard, Dino Rech, Veerle Dermaux-Msimang, Camille Legeai, David Lewis, Beverley Singh, Adrian Puren, Bertran Auvert

**Affiliations:** 1 CESP INSERM-UVSQ UMRS 1018, Villejuif, France; 2 Progressus, Johannesburg, South Africa; 3 National Institute for Communicable Diseases (NICD) of the National Health Laboratory Services (NHLS), Johannesburg, South Africa; 4 University of the Witwatersrand, Johannesburg, South Africa; 5 Hôpital Ambroise Paré, Assistance Publique-Hôpitaux de Paris, Boulogne, France; 6 Université Versailles Saint-Quentin-en-Yvelines, Versailles, France

**Keywords:** male circumcision, foreskin, uptake, acceptability, HIV-AIDS

## Abstract

**Background:**

To evaluate the knowledge, attitudes and beliefs about adult male circumcision (AMC), assess the association of AMC with HIV incidence and prevalence, and estimate AMC uptake in a Southern African community.

**Methods:**

A cross-sectional biomedical survey (ANRS-12126) conducted in 2007-2008 among a random sample of 1198 men aged 15 to 49 from Orange Farm (South Africa). Face-to-face interviews were conducted by structured questionnaire. Recent HIV infections were evaluated using the BED incidence assay. Circumcision status was self-reported and clinically assessed. Adjusted HIV incidence rate ratios (aIRR) and prevalence ratios (aPR) were calculated using Poisson regression.

**Results:**

The response rate was 73.9%. Most respondents agreed that circumcised men could become HIV infected and needed to use condoms, although 19.3% (95%CI: 17.1% to 21.6%) asserted that AMC protected fully against HIV. Among self-reported circumcised men, 44.9% (95%CI: 39.6% to 50.3%) had intact foreskins. Men without foreskins had lower HIV incidence and prevalence than men with foreskins (aIRR = 0.35; 95%CI: 0.14 to 0.88; aPR = 0.45, 95%CI: 0.26 to 0.79). No significant difference was found between self-reported circumcised men with foreskins and other uncircumcised men. Intention to undergo AMC was associated with ethnic group and partner and family support of AMC. Uptake of AMC was 58.8% (95%CI: 55.4% to 62.0%).

**Conclusions:**

AMC uptake in this community is high but communication and counseling should emphasize what clinical AMC is and its effect on HIV acquisition. These findings suggest that AMC roll-out is promising but requires careful implementation strategies to be successful against the African HIV epidemic.

## Background

The protective effect of adult male circumcision (AMC) on HIV acquisition has been reported in a review of epidemiological studies [1] and demonstrated by three randomized controlled trials conducted in Southern and Eastern Africa, which found that the risk of HIV acquisition among circumcised adult men was reduced by about 60% [2-4]. As a health intervention, AMC is predicted to be significantly life- and cost-saving in terms of averted HIV infections and related medical costs [5-8].

In 2007, WHO/UNAIDS recommended AMC as an important, additional intervention which should be delivered as part of a comprehensive HIV prevention package in communities with generalized HIV epidemics and low AMC prevalence [9]. Since this recommendation, efforts are being applied to roll-out safe and effective AMC services in several Eastern and Southern African countries [10-12].

A review of studies investigating the acceptability of AMC as an intervention against HIV among Sub-Saharan African communities not practicing male circumcision was conducted in 2006 [13]. AMC acceptability among men was defined as their willingness to undergo the procedure. This review reported medium-high to high acceptability of AMC, if performed safely and at minimal cost, of 65% (95% confidence interval (CI): 29% to 87%) among men for themselves. As this review of acceptability studies was conducted before all AMC trials results were known, higher levels of acceptability may be expected following the WHO/UNAIDS recommendation [9].

Little is known however about the extent to which AMC as an intervention against HIV would be actually taken up in these communities. As demonstrated by modeling studies [6,14,15], the uptake of AMC is a key condition for a successful roll-out because it will condition the impact of the intervention on the spread of HIV. The most frequently reported barriers to AMC uptake in African communities, which are cost and surgical safety [13], are addressed when providing free medicalised AMC. Nonetheless, other factors may facilitate or inhibit intention to undergo AMC, and their identification is necessary to refine outreach and communication strategies, design effective AMC delivery models, and optimize the impact of AMC interventions on the HIV epidemic.

The overall aim of this study was to conduct an operational study of AMC uptake in a South African community. Specifically, the objectives were to a) evaluate community knowledge, attitudes and beliefs about male circumcision, b) assess male circumcision's association with HIV incidence and prevalence in the community, c) identify the demographic, biomedical, social, behavioral, and knowledge factors associated with intention to undergo AMC and d) estimate the uptake of free medicalised AMC as an intervention against HIV.

## Methods

### Study context

The study (ANRS-12126) was conducted from October 2007 to April 2008 in the township of Orange Farm, located south of Johannesburg in the Gauteng province of South Africa. The first published randomized clinical trial on the effect of AMC on HIV acquisition was conducted in this community in 2002-2005 [2]. The township has an estimated population of 200,000 living in an area of about 50 km^2^. A study conducted in a neighboring, comparable township, estimated self-reported circumcision prevalence at 22.4%, and clinical circumcision prevalence (lack of foreskin) at 13%, with male circumcision being perceived positively [16]. HIV prevalence in the province is estimated at 15.2% among adults aged 15 to 49 [17].

### Study recruitment

Screening for the biomedical survey was conducted according to a method designed for a community-based cross-sectional study conducted in the same area [16]. Briefly, a random sample of 1680 households was selected from Statistics South Africa Enumerator Area aerial photographs. The survey was self-weighted by dividing the township into clusters of similar housing types. In each cluster, the number of households randomly selected depended on the total number of households and the average number of inhabitants per household. All men aged 15 to 49, who had slept in these households the night before the visit of the investigative team, were eligible for inclusion. Voluntary, written informed consent was required, in addition to parental consent for those aged under 18.

### Data collection

Each participant was interviewed face-to-face at the study site in his or her preferred language using an anonymous structured standardized questionnaire adapted from an instrument designed by UNAIDS [18]. The following data were collected: Background characteristics, including self-reported circumcision status; sexual behavior and condom use; attitudes towards HIV; knowledge, attitudes and beliefs towards AMC; intention to undergo free medicalised male circumcision from all self-reported uncircumcised participants.

### Counseling and HIV testing

Each interview was followed by an individual counseling session, which included general information about HIV and STI prevention, with a specific focus on the effect of AMC on HIV, emphasizing the partial protection of AMC against HIV acquisition and the need for consistent condom use. Participants were encouraged to undergo HIV testing, which was provided at the study site using rapid tests. Self-reported uncircumcised men were offered free medicalised AMC. Those who accepted the procedure received an AMC voucher with their name and photo.

### Genital examination

Male participants underwent a health examination performed by a trained male nurse during which their clinical circumcision status (presence or absence of foreskin) was assessed.

### Laboratory procedures

Each participant was asked to supply a venous blood sample (8 ml) for HIV and Herpes Simplex Virus 2 (HSV-2) testing. Samples were collected in plasma preparation tubes, centrifuged and harvested in aliquots (2 × 1.8 ml). A screening test (Genscreen HIV1/2 version 2, Bio-Rad, France) was performed on all aliquots. For reactive samples, a confirmatory test was run (Vironostika HIV Uni-Form II plus O, bioMérieux, Netherlands). If the sample reacted positively for both assays, a second confirmatory test was conducted (Murex HIV-1.2.O, Murex Biotech Ltd., UK). Plasma samples testing positive for HIV were retested using a HIV incidence assay (Calypte HIV-1 BED Incidence EIA (BED), Calypte Biomedical Corporation, USA), according to the manufacturer's protocol. HSV-2 testing was performed using the Kalon HSV-2 gG2 assay (Kalon Biological Ltd., UK).

### Management of STI and HIV-positive persons

Participants with symptomatic STIs were treated free of charge at the study site or at local health facilities. Individuals testing HIV positive were offered an immediate CD4 count at the study site. For CD4 counts of less than 200/ml, antiretroviral treatment (ART) was arranged in collaboration with the health facilities delivering ART in the community.

### AMC surgery

To undergo AMC surgery, willing men had to agree to follow the instructions provided by the medical team, especially abstaining from sexual activity for 6-weeks after being circumcised. Volunteers with contraindications for AMC surgery, such as allergy to anesthesia, hemophilia, bleeding disorders, genital ulceration, symptomatic STIs, signs of infections, abnormal genital anatomy or history of diabetes, were excluded. AMC surgeries were performed by trained medical doctors according to WHO surgical recommendations [19] using the forceps guided method, electrocautery, and sterilized disposable circumcision kits. The AMCs were standardized and performed using task-sharing by a medical team composed of five nurses and a medical circumciser, as described elsewhere [20]. After the procedure, participants were provided with analgesics for the relief of pain, given detailed postoperative instructions on wound care and management, including the mandatory 6-week abstinence from sexual activities, and asked to return to the centre for one follow-up visit, 2 to 4 days after surgery.

### Additional sample

To increase the power of the analyses testing the associations of reported and clinical male circumcision status with HIV incidence and prevalence, an additional random sample of 802 men aged 16 to 29 was surveyed one month after the end of the initial survey. These men were selected as described above and underwent the same procedures but a simplified questionnaire was used.

### Statistical Methods

Participants were compared by self-reported circumcision status and clinical circumcision status. For continuous data, medians and interquartile ranges (IQR) were computed, and significance testing was carried out using the Kruskal-Wallis test. Median and IQR of age at first sexual intercourse were computed using Kaplan-Meier survival analysis and compared between groups using the log-rank test. For categorical data, proportions were computed and compared between groups using Pearson's Chi square or Fisher exact tests, as applicable, and 95% confidence intervals (CI) were obtained by Bayesian calculations.

The comparison between self-reported circumcised men and self-reported uncircumcised men was performed among men aged 22 and older. In this age group, the median age at circumcision was 19 (IQR = 16-21), hence most of those who wanted to become circumcised were already circumcised. This prevented a dilution effect that could have occurred if younger men had been included since they could still become circumcised in the future.

Unadjusted odds ratios (OR) and adjusted OR (aOR) were computed using univariate and multivariate logistic regression analyses to assess the association of covariates with the following dichotomous variables: a) self-reporting as uncircumcised among all men aged 22 and older b) having an intact foreskin among all self-reported circumcised men and c) intending to undergo free and medicalised AMC among self-reported uncircumcised men. For the multivariate analyses, a forward stepwise procedure, with age and ethnic group being forced into the model, was used to select the significant covariates.

HIV incidence rates were calculated using the BED assay results with a cut-off value of 1.89, which corresponds to an assay window period of about 15 months, and with correction for misclassifications according to a published method [21]. Using Poisson regression, adjusted HIV incidence rate ratios (aIRR) and adjusted HIV prevalence ratios (aPR) were calculated, between a) self-reported circumcised men with foreskins and self-reported uncircumcised men with foreskins, and b) men clinically uncircumcised (with foreskins) and men clinically circumcised (without foreskin). We have also calculated the aIRR when using a cutoff of 1.51, corresponding to an assay window period of about 12 months. All analyses were adjusted on the relevant demographic and sexual behavior covariates listed in the data collection section above. To optimize these analyses, aIRR and aPR calculations were conducted among men aged 22 to 34, the age range in which HIV prevalence increases with age, and time since circumcision is at least two years. The estimated IRR were corrected for BED assay misclassifications. The details of the corrections are provided in the Additional file 1.

AMC uptake was calculated as the proportion of men who used the AMC vouchers to undergo AMC among all uncircumcised men aged 15 to 49.

All statistical analyses were performed using the statistical package SPSS version 8.0 for Windows (SPSS Inc., Chicago, IL, USA) and R version 2.10.1 [22].

### Ethics

Ethical clearance was granted by the Human Research Ethics Committee (Medical) of the University of the Witwatersrand on May 8th, 2007 (protocol study no.M070367).

## Results

### Characteristics of survey participants by self-reported circumcision status

The household and individual combined response rate was 73.9%. Among the 1198 male respondents, 334 (27.9%; 95%CI: 25.4% to 30.5%) self-reported as circumcised. Background characteristics, sexual behavior, attitudes towards HIV, and prevalence of HIV and HSV-2 are reported by self-reported circumcision status in Table 1. Multivariate analysis indicated that self-reported uncircumcised men were more likely to be aged 27 or older (aOR = 1.72; 95%CI: 1.15 to 2.56), more often from Zulu (traditionally non-circumcising) than Sotho (traditionally circumcising) ethnicity (aOR = 1.84; 95%CI: 1.22 to 2.77), more often single than ever married (aOR = 2.06; 95%CI: 1.20 to 3.55), more likely to have initiated sexual activity after the age of 16 (aOR = 1.55; 95%CI: 1.09 to 2.21), more often HIV-positive (aOR = 1.91; 95%CI: 1.20 to 3.03) and less likely to be aware of their HIV status (aOR = 0.65; 95%CI: 0.46 to 0.93). No association was found with key factors associated with increased risk of HIV acquisition, such as number of sexual partners and lack of consistent condom use with non-spousal partners.

**Table 1:** Survey participants' characteristics, by self-reported circumcision status

	Men aged 22 and over		
	Self-reported circumcised	Self-reported uncircumcised	***P*-value**^2^
**Sample size**	234	374	

**Background Characteristics**			

Age			
*Mean (median)*	29.1 (26)	29.9 (28)	0.07
*IQR*	24-33	24-34	
Ethnic group (%)			
*Sotho*	32.9	27.8	
*Zulu*	34.6	50.8	<0.001
*Other*	32.5	21.4	
Religion (%)			
*Christian*	36.3	35.3	0.09
*No religion*	44.0	51.1	
*Other*	19.7	13.6	
Education (%)			
*Grade 12 completed*	28.6	27.0	0.71
Occupation (%)			
*Employed*	57.7	58.6	
*Unemployed*	28.6	31.0	
*Scholar or student*	4.3	4.0	0.57
*Other*	9.4	6.4	
Marital status (%)			
*Ever married*	36.5	31.8	0.31
*Committed to someone*	46.8	47.1	
*Single*	16.7	21.1	
Initiation school attendance (%)			
	42.5	3.5	<0.001

**Reported sexual behavior**			

Ever had sexual intercourse (%)			
	99.6	98.7	0.41
Age at first sexual intercourse (year)			
*Mean (median)*	16.1 (16)	16.7 (16)	0.02
*IQR*	14.0-17.0	15.0-18.0	
Number of lifetime sexual partners^1^			
*Mean (median)*	16.2 (10)	13.8 (8)	0.19
*IQR*	5-20	4-15	
Number of sexual partners in the past 12 months^1^			
*Mean (median)*	2.8 (2)	2.5 (2)	0.04
*IQR*	1.0-3.0	1.0-3.0	
Ever used a condom^1 ^(%)			
	90.1	86.2	0.16
Consistent condom use in the past 12 months, with non-spousal partners (%)			
	26.6	34.5	0.08

**Attitudes towards HIV**			

Perceived risk of HIV infection (%)			
*No or small risk*	48.7	43.2	0.36
*Average or high risk*	32.5	37.5	
*No opinion*	18.8	19.3	
Aware of HIV status (%)			
	42.3	32.9	0.02

**Sexually transmitted infections**			

HIV-positive			
	15.0	25.1	0.003
HSV-2 positive			
	30.8	35.6	0.25

HSV-2: Herpes Simplex Virus 2

^1 ^Among those having had sexual intercourse

^2 ^*P-values *were obtained when comparing self-reported uncircumcised men and self-reported circumcised men using Kruskal-Wallis, Pearson's Chi square, Fisher's exact or log-rank test, as applicable

### Clinical circumcision status and HIV risk

Following genital examination, it was observed that 44.9% (95%CI: 39.6% to 50.3%) of self-reported circumcised men had intact foreskins, whereas 99.7% (95%CI: 99.1% to 99.9%) of self-reported uncircumcised men had foreskins. Self-reported circumcised men represented 14.8% (95%CI: 12.7% to 17.1%) of all men with foreskins. In multivariate analysis, among self-reported circumcised men, having an intact foreskin was associated with older age (*P*_linear trend _= 0.01), being of Zulu or Sotho ethnicity (aOR = 3.4; 95%CI: 1.90 to 6.09), having attended initiation school (aOR 7.7; 95%CI: 4.48 to 13.30) and being a scholar or a student (aOR = 3.58; 95%CI: 1.74 to 7.37).

**Figure 1:**
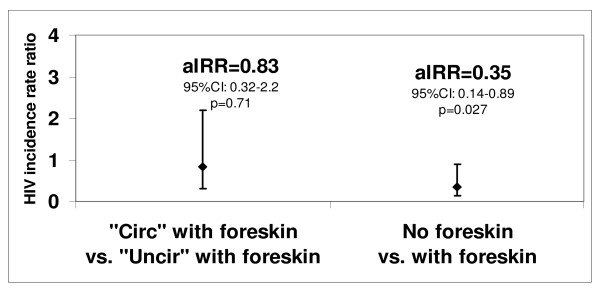
**Adjusted HIV incidence rate (aIRR) by self-reported male circumcision status and clinical circumcision status among men aged 22 to 34**. Self-reported uncircumcised and self-reported circumcised men are labeled "Uncir" and "Circ" on the figure, respectively. aIRR, 95% confidence intervals (CI) and p-values (p) were calculated using Poisson regression. Covariates were age, ethnic group, marital status, number of lifetime sexual partners, number of sexual partners in the past 12 months, consistent condom use with non spousal partners and HSV-2 status.

**Figure 2:**
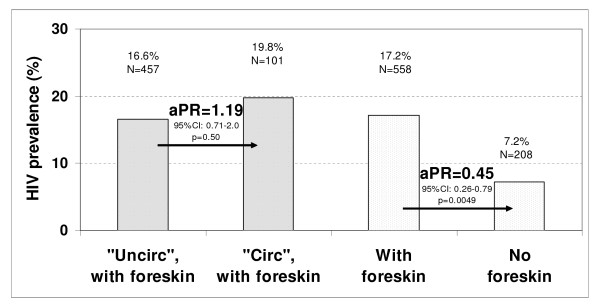
**Adjusted HIV prevalence rate (aPR) by self-reported male circumcision status and clinical circumcision status among men aged 22 to 34**. Self-reported uncircumcised and self-reported circumcised men are labeled "Uncir" and "Circ" on the figure, respectively. The two darker bars to the left represent men with foreskins, by reported circumcision status. The two lighter bars to the right represent all men in the sample, by clinical circumcision status. aPR, 95% confidence intervals (CI) and p-values (*P*) were obtained using Poisson regression. Covariates are the same as in Figure 1.

Clinically circumcised men had a mean time since circumcision of 8.8 years (median = 6.5 years; IQR: 4.5 years-11.5 years). Among these men, six were tested recent seroconverters and 193 were HIV-negative, corresponding to an HIV incidence of 0.022 per person-year. Among clinically uncircumcised men, the corresponding figures were 37, 462 and 0.056 per person-year. The IRR was 0.40 (95%CI: 0.16 to 0.98; *P *= 0.05). Among clinically circumcised men, HIV aIRR was about two-thirds lower and HIV aPR was more than half lower than among all other men (Figures 1 and 2). When using an assay window period of about 12 months, the aIRR was 0.30 (95%CI: 0.10 to 0.80), which is close to the value found with the 15 months assay window.

No differences in HIV incidence and prevalence between self-reported circumcised men with a foreskin and other uncircumcised men were detected. There was no significant variation of the protective effect of AMC with time since circumcision. On average, this effect increased the aIRR by 4.1% per year (95%CI: -4.1% to 11.6%, *P *_linear trend _= 0.27), corresponding to a non significant weaker effect.

### Knowledge, attitudes and beliefs towards AMC

Knowledge, attitudes and beliefs towards AMC, by reported circumcision status, are detailed in Table 2. Most respondents agreed that circumcised men could become HIV infected (92.6%; 95% CI: 91.0% to 94.0%) and needed to use condoms (90.0%; 95% CI: 88.2% to 91.6%), although 19.3% (95% CI: 17.1% to 21.6%) asserted that AMC protected fully against HIV. When compared with self-reported uncircumcised men, self-reported circumcised men were more likely to believe that women preferred circumcised men, that AMC increased sexual pleasure, that circumcised men did not need to use condoms and to report that their partners and families supported AMC.

**Table 2:** Knowledge, attitudes and beliefs towards adult male circumcision (AMC), by self-reported male circumcision status

	Men aged 22 and over		
	Self-reported circumcised	Self-reported uncircumcised	***P*-value**^1^
**Sample size**	234	374	

AMC protects fully against HIV (%)			

*Agree*	22.2	16.0	0.06
*Disagree*	61.5	61.5	
*Do not know*	16.2	22.5	

Most women prefer circumcised men (%)			

*Agree*	70.9	56.1	0.001
*Disagree*	10.3	14.4	
*Do not know*	18.8	29.4	

AMC increases sexual pleasure (%)			

*Agree*	68.4	36.1	< 0.001
*Disagree*	16.2	19.0	
*Do not know*	15.4	44.9	

Circumcised men do not need to use condoms for protection against HIV and other STIs (%)			

*Agree*	6.4	4.8	0.01
*Disagree*	91.0	86.6	
*Do not know*	2.6	8.6	

Circumcised men can become infected with HIV (%)			

*Agree*	93.2	90.9	0.13
*Disagree*	3.8	2.7	
*Do not know*	3.0	6.4	

My partner supports AMC (%)			

*Agree*	68.4	42.8	< 0.001
*Disagree*	9.8	22.2	
*Do not know*	21.8	35.0	

My family supports AMC (%)			

*Agree*	85.5	46.0	< 0.001
*Disagree*	10.3	37.7	
*Do not know*	4.3	16.3	

AMC is safe when carried by a doctor (%)			

*Agree*	87.2	90.6	0.41
*Disagree*	4.7	3.5	
*Do not know*	8.1	5.9	

I would prefer to have my male children circumcised (%)			

Yes	96.6	78.1	< 0.001
No	3.4	21.9	

STIs: sexually transmitted infections

^1 ^*P-values *were obtained using Pearson's Chi square or Fisher exact tests, as applicable.

### Intention to undergo AMC and AMC uptake

Among the 861 self-reported and clinically uncircumcised men, 699 (81.2%; 95%CI: 78.4% to 83.7%) stated that they would want to undergo AMC if it was free and performed by a doctor.

Among these men, the most frequently stated reasons for not being circumcised were pain (21.5%; 95%CI: 18.5% to 24.6%), AMC not being part of one's culture (12.6%; 95%CI: 10.3% to 15.2%), and the risks (10.0%; 95%CI: 7.9% to 12.4%) and costs (6.2%; 95%CI: 4.5% to 8.1%) associated with the procedure. A sizeable proportion of the respondents (22.5%; 95%CI: 19.5% to 25.6%) reported no specific reason.

In the multivariate analysis, intention to undergo AMC was associated with ethnic group, believing that medicalised AMC was safe and partner and family support of AMC (Table 3).

**Table 3:** Factors associated with intention to undergo free medicalised adult male circumcision (AMC) among self-reported uncircumcised men aged 22 and over

	Intention to undergo AMC % (N)	**Univariate Odds ratio**^**1 **^**(95%CI)**	**Adjusted Odds ratio**^**2 **^**(95%CI)**
**Background Characteristics**			

Age			
*Less than 27*	85.8 (162)	1	1
*27 and over*	76.6 (209)	0.54 (0.31 to 0.93) *P *= 0.03	0.63 (0.32 to 1.14) *P *= 0.10
Ethnic group			
*Sotho*	89.4 (104)	1	1
*Zulu*	77.2 (189)	0.40 (0.23 to 0.82) *P *= 0.01	0.36 (0.17 to 0.79) *P *= 0.01
*Other*	76.9 (78)	0.39 (0.17 to 0.89) *P *= 0.03	0.33 (0.13 to 0.80) *P *= 0.02
Religion			
*Christian*	81.1 (132)	1	NS
*No religion*	78.2 (188)	0.83 (0.48 to 1.46) *P *= 0.53	
*Other*	88.2 (51)	1.70 (0.66 to 4.61) *P *= 0.25	
Education: grade 12 completed			
*No*	80.8 (217)	1	NS
*Yes*	80.0 (100)	0.95 (0.53 to 1.70) *P *= 0.86	
Occupation			
*Employed*	78.7 (216)	1	NS
*Unemployed*	82.8 (96)	1.28 (0.73 to 2.32) *P *= 0.38	
*Other*	84.6 (39)	1.50 (0.59 to 3.84) *P *= 0.40	
Marital status			
*Ever married*	76.3 (118)	1	NS
*Committed to someone*	79.4 (175)	1.21 (0.69 to 2.09) *P *= 0.52	
*Single*	89.7 (78)	2.69 (1.23 to 6.27) *P *= 0.02	

**Reported sexual behavior**			

Number of lifetime sexual partners			
*Less than 8*	81.2 (181)	1	NS
*8 or more*	80.0 (190)	0.93 (0.55 to 1.50) *P *= 0.77	
Linear trend	NA	1.00 (0.98 to 1.01) *P *= 0.60	
Number of sexual partners in the past 12 months			
*Less than 2*	81.2 (181)	1	NS
*2 or more*	79.8 (188)	0.91 (0.54 to 1.48) *P *= 0.73	
Linear trend	NA	0.97 (0.91 to 1.03) *P *= 0.30	
Ever used a condom			
*Yes*	81.6 (315)	1	NS
*No*	75.0 (56)	0.68 (0.35 to 1.31) *P *= 0.39	
Consistent condom use in the past 12 months, with non-spousal partners			
*Yes*	81.3 (96)	1	NS
*No*	80.4 (179)	0.95 (0.50 to 1.79) *P *= 0.87	

**Attitudes towards HIV and awareness of HIV status**			

Perceived risk of infection with HIV			
*No or small risk*	81.1 (159)	1	NS
*Average or high risk*	82.7 (139)	1.11 (0.62 to 2.02) *P *= 0.72	
Aware of HIV status			
*Yes*	81.0 (121)	1	NS
*No*	80.4 (250)	0.96 (0.55 to 1.70) *P *= 0.89	

**Knowledge, attitudes and beliefs towards AMC**			

AMC protects fully against HIV			
*Disagree*	80.3 (229)	1	NS
*Agree*	86.2 (58)	1.50 ( 0.68 to 3.40) *P *= 0.31	
Most women prefer circumcised men			
*Disagree*	71.7 (53)	1	NS
*Agree*	84.6 (208)	2.20 (1.10 to 4.40) *P *= 0.032	
AMC increases sexual pleasure			
*Disagree*	78.9 (71)	1	NS
*Agree*	86.4 (132)	1.70 (0.80 to 3.60) *P *= 0.17	
Circumcised men need to use condoms for protection against HIV and other STIs			
*Disagree*	72.2 (18)	1	NS
*Agree*	82.6 (322)	1.80 (0.63 to 5.30) *P *= 0.27	
Circumcised men can become infected with HIV			
*Disagree*	100 (10)	1	NS
*Agree*	80.4 (337)	NC	
My partner supports AMC			
*Disagree*	63.4 (82)	1	1
*Agree*	87.3(158)	4.03 (2.10 to 7.60) *P *< 0.001	2.59 (1.20 to 5.61) *P *= 0.02
My family supports AMC			
*Disagree*	68.8 (141)	1	1
*Agree*	90.6 (170)	4.41 (2.33 to 8.20) *P *< 0.001	2.92 (1.41 to 6.03) *P *= 0.005
AMC is safe when it is carried out by a doctor			
*Disagree*	38.5 (13)	1	1
*Agree*	83.6 (336)	8.18 (2.61 to 25.9) *P *< 0.001	11.01 (3.10 to 39.04) *P *< 0.001

**Sexually Transmitted Infection**			

HIV infection			
*No*	81.6 (277)	1	NS
*Yes*	77.7 (94)	0.78 (0.44 to 1.42) *P *= 0.41	
HSV-2 infection			
*No*	82.8 (239)	1	NS
*Yes*	76.5 (101)	0.67 (0.39 to 1.10) *P *= 0.14	

^1 ^Obtained using logistic regression

^2 ^Obtained using forward stepwise logistic regression with all the variables indicated in this table

N: Sample size

CI: Confidence interval

*P*: P-value

NS: Not selected by the forward stepwise logistic regression

NA: Not available

NC: Not calculable

STI: sexually transmitted infection

HSV-2: Herpes Simplex Virus 2

Among men reporting intention to undergo AMC, 72.4% (506/699) were circumcised through the study. Uptake of AMC was 58.8% (506/861; 95%CI: 55.4% to 62.0%).

## Discussion

This operational study of AMC uptake in a South African community indicates that when offered free medicalised AMC, more than half of self-reported uncircumcised men choose to become circumcised. Furthermore, the study reveals that about half of self-reported circumcised men in the study had foreskins, and that when considering only men with foreskins, HIV prevalence did not differ between self-reported circumcised men and self-reported uncircumcised men. Conversely, the reported protective effect of clinical circumcision on HIV acquisition was higher than what was reported in the three male circumcision trials [2-4]. The study also established that most men in the community had a fairly good knowledge of AMC and its association with HIV acquisition, despite some misconceptions, and suggested that intention to undergo AMC was associated with social factors. No association was found between self-reported circumcision status and risky sexual behavior. Furthermore, men willing to become circumcised were neither more nor less likely to be HIV-positive or at higher or lower risk of acquiring HIV than men who were not willing to undergo the procedure. Lastly, no evidence of a variation of the protective effect of AMC on HIV incidence with time since circumcision was found.

It is not possible to compare the uptake reported here with other findings since this is, to the best of our knowledge, the first study on AMC uptake conducted among a random sample representative of the general population. However, another South African study has reported an uptake of 33%, lower than the present estimate, in a non-random AMC study nested in an HIV efficacy trial [23].

This study has two main limitations. The first limitation is that it was only possible to determine the characteristics of participants who reported intention to undergo AMC, and not of those who actually underwent surgery, due to the way anonymous data were collected. However, more than 70% of the men who reported intention to undergo AMC were circumcised. A second limitation is that this study was conducted in the township where the first AMC trial was conducted, which may have influenced the decision to undergo AMC and could have enhanced community knowledge about the association between AMC and HIV acquisition. It is unlikely because a survey conducted in 2008 among a random sample of male residents found that only 2.1% knew the results of the AMC trial [20]. Nonetheless, even if Orange Farm is considered a typical South African township, some caution should be used when generalizing these results to other South African communities or to other countries.

One of the most interesting findings of this study is the fact that almost half of self-reported circumcised men had in fact an intact foreskin. This is most probably due to the initiation rituals which are customarily practiced in Southern and Eastern Africa. In South Africa, and this may also be true elsewhere, the initiation rituals may or may not involve the actual removal of the foreskin [24,25]. Hence, men having undergone such initiation rituals, usually around puberty, may call themselves "circumcised", even if their foreskin is intact. This may also explain the apparent contradictions in knowledge, attitudes & beliefs about AMC and "circumcised" men found in this study.

The study findings, along with other examples of AMC roll-out interventions which are ongoing in Kenya, Botswana, Swaziland, Zambia and Zimbabwe [12], provide evidence that a satisfactory uptake can be expected from the AMC scale-up interventions that are on-going in other countries of Southern and Eastern Africa [10]. Furthermore, the findings indicate that such interventions are likely to reach men from the general population and not just those who are at higher or lower risk of HIV infection. Therefore, if a high uptake is obtained, the effect of AMC roll-out on HIV prevalence at population level may be substantial after some years, as predicted by modeling studies [6,14,26].

The study has some important implications for the planning of AMC roll-out. First, men who think that they are circumcised but who are not in reality must be reached. A possibility would be to include in the communication and information documentation photos and diagrams that illustrate what a circumcised penis looks like. A randomized trial aiming to assess methods to improve the self-reporting of male circumcision status among men and their partners was conducted in 2010 in Swaziland and Zambia [12]. The upcoming results of this trial will be helpful to identify the best approach. Secondly, it is likely that AMC roll-out interventions will require extensive communication campaigns to explain what clinical AMC is and its effect on HIV acquisition. Indeed, in Orange Farm, despite a high acceptability of male circumcision and the availability of clinical AMC in the community at a cost of about 40 Euros in most local medical practices, only about 15% of the men are clinically circumcised. In the present study, to achieve the reported uptake, free medicalised AMC was offered to each eligible man during individual counseling sessions. It is unknown whether such individual contacts will still be required once national AMC campaigns are launched. Thirdly, the AMC promotion campaigns should target both primary and secondary audiences. Indeed, the importance of family and partners support of AMC on intention to undergo clinical AMC is a noteworthy finding. Fourthly, the partial protective effect of AMC should be central to communication and counselling strategies. Although current knowledge about the effect of AMC on HIV acquisition is fairly good among men from the general population, there is still a sizeable proportion who think that circumcised men are not at risk of getting HIV and do not need to use condoms for protection against HIV and other STIs. Lastly, what AMC campaigns report about issues of sexual pleasure and partners' preference may have some implications on AMC uptake. In the present study, some men, in particular those who are self-reporting as circumcised, have the beliefs that AMC increases sexual pleasure and that women might prefer circumcised men. However, scientific evidence on this issue has not been established [27,28].

## Conclusion

This study demonstrates that AMC roll-out is a promising intervention against the HIV epidemic in Africa but that it will require careful design and comprehensive communication strategies to be successful.

## Competing interests

The authors declare that they have no competing interests.

## Authors' contributions

PL and BA contributed equally to this work. DT collected the data. DR performed the male circumcisions. PL, CL and BA analyzed the data. VDM, DL, BS and AP analyzed the biological samples. All the authors contributed to, and have read and approved the final version of the manuscript.

BA and PL accept full responsibility for the work and the conduct of the study, the integrity of the data and the accuracy of the data analysis. They had full access to the data and controlled the decision to publish.

## Pre-publication history

The pre-publication history for this paper can be accessed here:

http://www.biomedcentral.com/1471-2334/11/253/prepub

## Supplementary Material

Additional File 1 **Calculating HIV incidence and multivariate HIV incidence rate ratio using the BED assay results**. This file provides mathematical formulas, as well as calculation details, which were used for the computation of the HIV incidence rate and the multivariate HIV incidence rate ratio, using the BED assay results with corrections for misclassifications. Click here for file
