# Supplementary material for: Adult male circumcision as an intervention against HIV: An operational study of uptake in a South African community (ANRS 12126)
Source: BMC Infect Dis. 2011 Sep 26;11:253. doi: 10.1186/1471-2334-11-253 (PMC3192707; doi:10.1186/1471-2334-11-253)
Supplement: Additional File 1 — Calculating HIV incidence and multivariate HIV incidence rate ratio using the BED assay results. This file provides mathematical formulas, as well as calculation details, which were used for the computation of the HIV incidence rate and the multivariate HIV incidence rate ratio, using the BED assay results with corrections for misclassifications. [file 1471-2334-11-253-S1.DOC]

**Additional File 1**

**Word count:** 754

**Calculating HIV incidence and multivariate HIV incidence rate ratio using the BED assay results**

**Introduction**

The BED HIV-1 Capture EIA (BED assay) identifies recent HIV infections by detecting the proportion of HIV-specific immunoglobin G (which increases as a function of time since infection) present in total serum immunoglobin G after seroconversion [1]. This proportion is measured as a normalized optical density (ODn). Samples which are below a determined ODn “cut-off” value are identified as “tested recent seroconversions”. The mean duration between seroconversion and the time for the ODn to exceed the cut-off is called the BED assay window period, also defined as the duration during which an individual is identified as “recent seroconverter” by the assay [1]. Because the BED assay tends to misclassify a proportion of non-recent infections as recent seroconversions, three calibration parameters have been introduced by McDougal et al. [2], who considered three time intervals. The first interval is before HIV testing and equal in duration to the window period. The second interval is immediately before the first interval and equal in duration to the window period. The third interval is the period before the second interval. The sensitivity (S_e_) is defined as the ability of the test to detect true recent seroconverters during the first interval. The specificity is defined as the ability of the test to identify true non-recent seroconverters in the second interval (short-term specificity; ρ_1_) and in the third interval (long-term specificity; ρ_2_).

**BED window period and short-term specificity**

Let C_BED_ be the BED cut-off value and W the BED window period in months. We define the relationship between C_BED_ and W as follows: When following up a group of HIV-negative individuals participating in a study for a period of 2W before testing, C_BED_ is selected such that the number of tested recent seroconverters is equal to the number of participants who became HIV-positive in the period W before testing. Using the McDougal et al. terminology [2], this definition implies that the sensitivity is equal to the short-term specificity (S_e_ = ρ_1_). Using another relationship between C_BED_ and W, McWalter et al. have obtained sensitivity equals to short-term specificity plus one minus long-term specificity [3].

From the Orange Farm male circumcision data, we obtained a window period of 185 days (95%CI: 146 to 227) when using a BED cut-off of 0.80 [4]. We also calculated C_BED_ for several W varying from three months to 18 months in 3-month increments. The results indicated that W and the long-term specificity varied linearly with C_BED_ according to the following equations: W =8.0914 C_BED_ and ρ_2_=1-0.057448 C_BED_. It was also found that the sensibility was 0.87.

**Basic equations**

We labeled I_a_, I_b_ and I_c_ the numbers of new infections which occurred during the first, second and third interval, respectively. The sum I_a_+I_b_+I_c_ is the number (N_+_) of HIV-positive individuals at t=0. We called N_a_, N_b_ and N_c_ the number of individuals tested recent seroconverters among those infected during each interval. The sum Na+Nb+Nc is the number (N_TR_) of those tested recent seroconverters at time t=0. We called N_R_ the real number of recent infections. We assumed, as it is commonly done, that I_a_=I_b_ [2,3].

The following system 1 links the parameters and the variables.

$\text{ρ}_{2}=1-\frac{\text{N}_{\text{c}}}{\text{I}_{\text{c}}}$; $\text{ρ}_{1}=1-\frac{\text{N}_{\text{b}}}{\text{I}_{\text{b}}}$; $\text{Se}=\frac{\text{N}_{\text{a}}}{\text{I}_{\text{a}}}$; $\text{S}_{\text{e}}=\text{ρ}_{1}$

$\text{N}_{\text{TR}}=\text{N}_{\text{a}}+\text{N}_{\text{b}}+\text{N}_{\text{c}}$;$\text{N}_{+}=\text{Ia}+\text{Ib}+\text{Ic}$;$\text{N}_{\text{R}}=\text{I}_{\text{a}}$; $\text{I}_{\text{a}}=\text{I}_{\text{b}}$

It follows that $\text{I}_{\text{a}}=\frac{\text{N}_{\text{TR}}-(1-\text{ρ}_{2}\text{)N}_{+}}{2\text{ρ}_{2}-1}$

We denoted x (y) the probability that a participant tested (not) recent seroconverter was HIV negative at time t-W. It followed that x and y were given by the following formulae:

$$\text{x}=\frac{\text{N}_{\text{a}}}{\text{N}_{\text{TR}}}=\text{I}_{\text{a}}\frac{\text{S}_{\text{e}}}{\text{N}_{\text{TR}}}=\left(\frac{\text{S}_{\text{e}}}{\text{N}_{\text{TR}}}\right)\left(\frac{\text{N}_{\text{TR}}-(1-\text{ρ}_{2}\text{)N}_{+}}{2\text{ρ}_{2}-1}\right)$$

$$\text{y}=\frac{\text{I}_{\text{a}}\text{-N}_{\text{a}}}{\text{N}_{+}\text{-N}_{\text{TR}}}=\text{I}_{\text{a}}\frac{\text{1-S}_{\text{e}}}{\text{N}_{+}\text{-N}_{\text{TR}}}=\left(\frac{\text{1-S}_{\text{e}}}{\text{N}_{+}\text{-N}_{\text{TR}}}\right)\left(\frac{\text{N}_{\text{TR}}-(1-\text{ρ}_{2}\text{)N}_{+}}{2\text{ρ}_{2}-1}\right)$$

**Estimation of HIV incidence rate**

If we had observed the HIV status of the participants at the end of a period equal to W, with all participants HIV-negative at the beginning of the period, HIV status could have been modeled using an exponential proportional hazards model, in which the baseline hazard was considered constant during W. This theoretical model could be implemented by running a Poisson log-linear model to obtain IR and IRR with or without adjustment for cofactors [6-8]. To analyze the data, we used the same model, with a weight of 1 for HIV-negative individuals, weights of *x* for those tested recent seroconverters, and weights of *y* for those tested not recent seroconverters. In addition, we used offsets of $\log(\frac{\text{W}}{12})$ for HIV-negative participants and of $\log(\frac{\text{W}}{24})$ for those HIV- positive.

**Estimation of HIV incidence rate ratio**

The HIV incidence rate ratio (IRR) is the ratio of the HIV incidence rates of the two groups, here denoted group A and group B. If the parameters (sensibility and specificities) are the same for the two groups, the IRR can be written as follows:

$$\text{IRR}=\frac{\left[\frac{\text{xN}_{\text{TR}}+\text{y(N}_{+}-\text{N}_{\text{TR}}\text{)}}{\text{WN}_{-}+\frac{\text{W}}{2}\text{(xN}_{\text{TR}}+\text{y(N}_{+}-\text{N}_{\text{TR}}\text{))}}\right]_{\text{A}}}{\left[\frac{\text{xN}_{\text{TR}}+\text{y(N}_{+}-\text{N}_{\text{TR}}\text{)}}{\text{WN}_{-}+\frac{\text{W}}{2}\text{(xN}_{\text{TR}}+\text{y(N}_{+}-\text{N}_{\text{TR}}\text{))}}\right]_{\text{B}}}=\frac{\left[\frac{\frac{\text{N}_{\text{TR}}-(1-\text{ρ}_{2}\text{)N}_{+}}{2\text{ρ}_{2}-1}}{\text{N}_{-}+\frac{\text{N}_{\text{TR}}-(1-\text{ρ}_{2}\text{)N}_{+}}{2(2\text{ρ}_{2}-1)}\text{)}}\right]_{\text{A}}}{\left[\frac{\frac{\text{N}_{\text{TR}}-(1-\text{ρ}_{2}\text{)N}_{+}}{2\text{ρ}_{2}-1}}{\text{N}_{-}+\frac{\text{N}_{\text{TR}}-(1-\text{ρ}_{2}\text{)N}_{+}}{2(2\text{ρ}_{2}-1)}\text{)}}\right]_{\text{B}}}$$

, which is independent of the sensibility and of W.

**REFERENCES**

1. Barnighausen T, McWalter TA, Rosner Z, Newell ML, Welte A. HIV incidence estimation using the BED capture enzyme immunoassay: systematic review and sensitivity analysis. *Epidemiology* 2010,**21**:685-697.

2. McDougal JS, Parekh BS, Peterson ML, Branson BM, Dobbs T, Ackers M*, et al.* Comparison of HIV type 1 incidence observed during longitudinal follow-up with incidence estimated by cross-sectional analysis using the BED capture enzyme immunoassay. *AIDS Res Hum Retroviruses* 2006,**22**:945-952.

3. McWalter TA, Welte A. A comparison of biomarker based incidence estimators. *PLoS One* 2009,**4**:e7368.

4. Fiamma A, Lissouba P, Oliver A, Singh B, Laeyendecker O, Quinn T*, et al.* Can HIV incidence testing be used for evaluating HIV intervention programs? A reanalysis of the Orange Farm male circumcision trial. *BMC Infectious Diseases* 2010,**10:137**.

5. McDougal JS. BED estimates of HIV incidence must be adjusted. *Aids* 2009,**23**:2064-2065; author reply 2066-2068.

6. Frome EL. The analysis of rates using Poisson regression models. *Biometrics* 1983,**39**:665-674.

7. Berry G. The analysis of mortality by the subject-years method. *Biometrics* 1983,**39**:173-184.

8. Holford TR. The analysis of rates and of survivorship using log-linear models. *Biometrics* 1980,**36**:299-305.
